# Potent antibiotic design via guided search from antibacterial activity evaluations

**DOI:** 10.1093/bioinformatics/btad059

**Published:** 2023-01-27

**Authors:** Lu Chen, Liang Yu, Lin Gao

**Affiliations:** School of Computer Science and Technology, Xidian University, Xi’an 710071, Shaanxi, China; School of Computer Science and Technology, Xidian University, Xi’an 710071, Shaanxi, China; School of Computer Science and Technology, Xidian University, Xi’an 710071, Shaanxi, China

## Abstract

**Motivation:**

The emergence of drug-resistant bacteria makes the discovery of new antibiotics an urgent issue, but finding new molecules with the desired antibacterial activity is an extremely difficult task. To address this challenge, we established a framework, MDAGS (Molecular Design via Attribute-Guided Search), to optimize and generate potent antibiotic molecules.

**Results:**

By designing the antibacterial activity latent space and guiding the optimization of functional compounds based on this space, the model MDAGS can generate novel compounds with desirable antibacterial activity without the need for extensive expensive and time-consuming evaluations. Compared with existing antibiotics, candidate antibacterial compounds generated by MDAGS always possessed significantly better antibacterial activity and ensured high similarity. Furthermore, although without explicit constraints on similarity to known antibiotics, these candidate antibacterial compounds all exhibited the highest structural similarity to antibiotics of expected function in the DrugBank database query. Overall, our approach provides a viable solution to the problem of bacterial drug resistance.

**Availability and implementation:**

Code of the model and datasets can be downloaded from GitHub (https://github.com/LiangYu-Xidian/MDAGS).

**Supplementary information:**

Supplementary data are available at *Bioinformatics* online.

## 1 Introduction

Bacterial drug resistance has led to an increasing number of health, social and economic problems that are becoming more prominent globally (Sugden *et al.*, 2016). Studies have shown that by 2050, if the global problem of antimicrobial resistance (AMR) is not addressed, 10 million people will die from AMR annually (Mulpuru *et al.*, 2021; Nieuwlaat *et al.*, 2021). The development of new antibiotics is typically abandoned due to unresolved regulatory issues and lack of commercial appeal (Årdal *et al.*, 2020). Furthermore, industry experts have analyzed unsuccessful antibacterial programs and found that high-throughput screening of synthetic chemicals often fails to find promising compounds with expected properties (Brown and Wright, 2016). Therefore, identifying and developing novel antibiotic drugs remains very challenging (Ismail *et al.*, 2021; Li *et al.*, 2021; Miethke *et al.*, 2021; Murray *et al.*, 2022; Theuretzbacher *et al.*, 2020).

In recent years, computational methods, especially deep learning, have emerged as a promising avenue for drug discovery by combining domain knowledge and data-driven learning (Button *et al.*, 2019; Camacho *et al.*, 2018; Hoffman *et al.*, 2022; Joshi *et al.*, 2021; Kotsias *et al.*, 2020; LeCun *et al.*, 2015; Lv *et al.*, 2022; Su *et al.*, 2019; von Lilienfeld and Burke, 2020). Among them, deep generative methods have been shown to be ideal for drug candidate discovery (Gómez-Bombarelli *et al.*, 2018; Putin *et al.*, 2018; Ru *et al.*, 2020; Sanchez-Lengeling and Aspuru-Guzik, 2018; Segler *et al.*, 2018b; Shaker *et al.*, 2021). In view of this, deep generative methods can be applied to generate ideal antibiotic molecules, thereby increasing the success rate of new antibiotic discovery and reducing the cost in the discovery process. To date, the related works of deep generative methods that have been reported in industry and academia (Dao *et al.*, 2021; Zulfiqar *et al.*, 2022) can be divided into generation-based and guide optimization-based, which are not mutually exclusive.

In generation-based work, many models represent a molecule with the corresponding simplified molecular input line entry specification (SMILES) notation so that the molecule generation task is transformed into a sequence-to-sequence generation task (Born *et al.*, 2021; Krishnan *et al.*, 2021; Lee *et al.*, 2021; Olivecrona *et al.*, 2017; Popova *et al.*, 2018; Segler *et al.*, 2018a; Wang *et al.*, 2021). However, this approach often requires large-scale pretraining, and grammatical errors will often lead to the generation of invalid SMILES, which cannot be converted into realistic molecular structures. There are also models that are represented as molecular graphs so that the task of generating molecules is transformed into a graph-to-graph generation task. Graph-based generation methods are further divided into autoregressive(Li *et al.*, 2018) and non-autoregressive (De Cao and Kipf, 2018; Jin *et al.*, 2018; Kwon *et al.*, 2019; Maziarka *et al.*, 2020; Simonovsky and Komodakis, 2018) generation. Autoregressive generation generates one atom at a time, borrowing ideas from sequence generation, and similarly, this method of generation also leads to chemically invalid intermediate graphs. Non-autoregressive generation generates the entire graph at once; this method has also been proven to be feasible, but its disadvantage is that it requires an additional graph matching process (Kwon *et al.*, 2019; Simonovsky and Komodakis, 2018).

In guided optimization-based work, the optimization can be in the generated molecular map or SMILES space or in the encoder–decoder latent space. For the former, the combination of a generative model and a property prediction model or scoring function for reinforcement learning is a common paradigm (Born *et al.*, 2021; De Cao and Kipf, 2018; Krishnan *et al.*, 2021; Lee *et al.*, 2021; Olivecrona *et al.*, 2017; Popova *et al.*, 2018; Segler *et al.*, 2018a; Wang *et al.*, 2021), but when the generative model is more complex, the generated results are poor since the prediction or scoring results cannot be efficiently fed back to the generative model (Wang *et al.*, 2021). For the latter, many models utilize different optimization strategies or sampling strategies for molecule generation (Button *et al.*, 2019; Chen *et al.*, 2021; Das *et al.*, 2021; Godinez *et al.*, 2022; Griffiths and Hernández-Lobato, 2020; Hoffman *et al.*, 2022; Jiménez-Luna *et al.*, 2019; Skalic *et al.*, 2019; You *et al.*, 2018).

In this article, we develop a novel generative method that combines attribute prediction models with an efficient guided search strategy to optimize and generate potent antibiotic molecules with expected antibacterial activity. Inspired by Graph2Seq models in the natural language domain (Tu and Coley, 2021; Xu *et al.*, 2018), we employ graph-to-sequence generation to exploit the advantages and avoid the disadvantages of existing sequence-to-sequence and graph-to-graph generation methods. We also decouple the encoder and decoder to reduce the complexity of the model. Furthermore, the encoder and attribute prediction model (Yang *et al.*, 2019) are jointly trained to learn a latent attribute space—the antibacterial activity space. In this latent space, a guided search based on attribute evaluation (Yang *et al.*, 2020) is used to guide the exploration of candidate antibiotic molecules in a meaningful direction (enhancing growth inhibition). We applied this method to generate antibiotic molecules with enhanced antibacterial activity compared to existing antibiotics, and these generated molecules achieved the greatest similarity to the expected functional antibiotic, although we did not explicitly constrain the similarity. Our approach combined a designed antibacterial activity space with guided searches to produce antibiotic molecules with enhanced inhibition for the first time. We believe our work will contribute to the development of new antibiotics.

## 2 Materials and methods

### 2.1 Data sources

#### 2.1.1 Drugs and natural products

We obtained the antibiotic and natural product dataset from Stokes *et al.* (2020) and obtained a primary training set containing 2560 compounds, of which 1760 compounds were screened for growth inhibition against *Escherichia coli BW25113* (Zampieri *et al.*, 2017) using a widely available US Food and Drug Administration-approved drug library. Another 800 are natural products isolated from plant, animal and microbial sources. After deduplication, 2334 compounds remained. For convenience, we call these 2334 compounds the main dataset. The mean inhibitory concentration—Mean_Inhibition (averaged over two rounds of experiments)—is a measure of the compound’s growth-inhibitory effect on *E.coli BW25113*. The distribution of growth inhibition values in the main dataset is shown in Supplementary Figure S1.

#### 2.1.2 MOSES dataset

The MOSES set is based on the ZINC (Irwin and Shoichet, 2005) Clean Leads collection. It contains a total of 4 591 276 molecules, filtered by molecular weight (MW) in the range 250–350 Da, with no more than seven rotatable bonds and XlogP≤3.5. The set excludes molecules containing charged atoms or atoms other than C, N, S, O, F, Cl, Br, H or cycles of more than eight atoms. The molecules were filtered via two filters—medicinal chemistry filters and PAINS filters (Polykovskiy *et al.*, 2020). The dataset contains 1 936 962 molecular structures. For experiments, the dataset is divided into training, test and scaffold test sets containing ∼1.6 M, 176 k and 176 k molecules, respectively.

#### 2.1.3 GuacaMol dataset

The GuacaMol dataset is derived from the ChEMBL 24 database (Mendez *et al.*, 2019). The advantage of ChEMBL is that it contains only molecules that have been synthesized and tested against biological targets (Brown *et al.*, 2019; Ru *et al.*, 2022). The dataset is divided into training, validation and test sets containing ∼1.3 M, 80 k and 240 k molecules, respectively.

### 2.2 Our MDAGS model

**Fig. 1.**
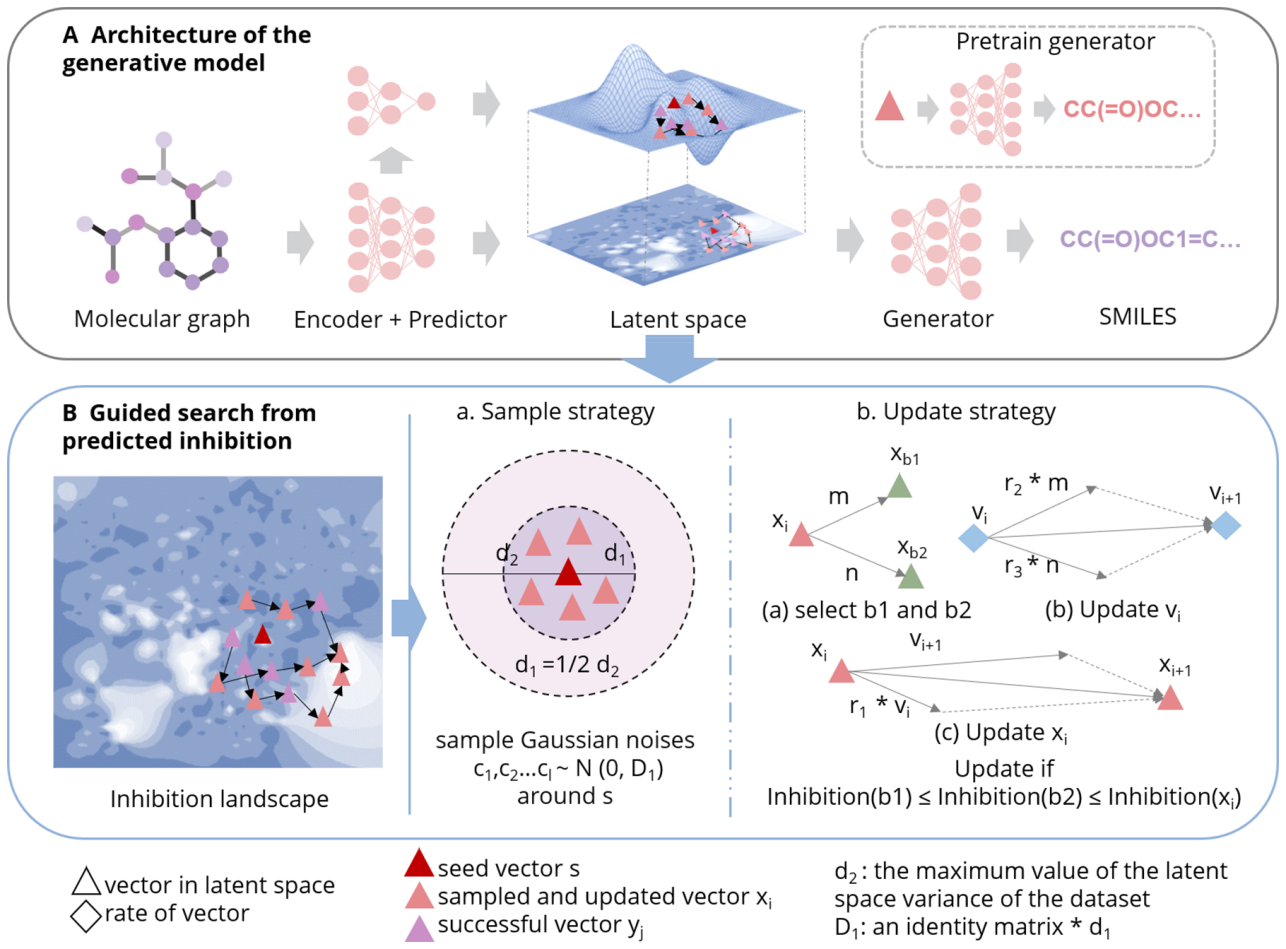
Overview of MDAGS. (**A**) The encoder and the predictor are jointly trained to obtain the latent vector representation of the input molecular graph and the predicted growth inhibition value, respectively, and the combination of the two constructs the potential growth inhibition space. The generator generates SMILES corresponding to the latent space optimization result after being pretrained with a large number of unlabeled molecules. (**B**) Latent space optimization consists of two parts: (i) sampling and (ii) updating. A given seed molecule is sampled and the sampled molecule is updated to find the molecule with the global minimum growth inhibition. Molecules satisfying the growth inhibition threshold during sampling and updating are considered successful molecules and are generated

The overall framework of the MDAGS model is shown in Figure 1.

#### 2.2.1 Architecture of the generative model

**Encoder–predictor for learning inhibition space.** We apply the directed message-passing neural network Chemprop (Yang *et al.*, 2019) to learn the inhibition space. The model consists of two parts: an encoder and a predictor. The encoder uses a directed message-passing mechanism to convert the molecular graph into a continuous latent space vector. The atomic features and edge features corresponding to the molecular graph are listed in Supplementary Table S1. By giving directions to the edges of the molecular graph and updating iterative messages based on the edges, unnecessary loops and jitter are avoided. During each iteration, the molecular graph substructure information is passed through the edges, and the node features are updated with the edge features (sum of the bond features). After multiple rounds of iterations, both edge and node features contain larger molecular graph substructure information and even global information, and the node feature aggregation (sum of the atom hidden states) can obtain the representation of the molecular graph. The equations for message passing and updating are:

(1) $$h_{ab}^{0}=\alpha (W_{i}[x_{a},e_{ab}]),$$

(2) $$m_{ab}^{t+1}=\sum_{c\in \left\{N(a)\backslash b\right\}}h_{ca}^{t},$$

(3) $$h_{ab}^{t+1}=\alpha (h_{ab}^{0}+W_{k}m_{ab}^{t+1}),$$

(4) $$m_{a}=\sum_{b\in \left\{N(a)\right\}}h_{ab}^{T},$$

(5) $$h_{a}^{}=\alpha (W_{y}[x_{a},m_{a}]),$$

where $h_{ab}^{t}$ represents hidden states and $m_{ab}^{t}$ represents messages, $x_{a}^{}$ represents the atom features and $e_{ab}^{}$ represents the bond features, $W_{i}\in R^{h*h_{i}}$ and $W_{k},W_{y}\in R^{h*h_{}}$ are learned matrixes, α is the activation function ReLU, [⋅,⋅] is concatenation and t∈{1,2⋅⋅⋅T} is the length of the message-passing step. And the readout phase operates according to

(6) $$h=\sum_{a\in G}h_{a}^{}.$$

The predictor is a feed-forward network (FFN) that predicts the growth inhibition value corresponding to the latent space vector

(7) y=f(h).

A latent property (growth inhibition) space of molecular graphs is learned by joint training of the encoder and predictor and optimized based on this space.

**Generator.** Inspired by the work of Bagal *et al.* (2022), we train a GPT model as the generator to generate molecular SMILES sequences corresponding to the latent space optimization results (see Section 2.2.2 for the optimization procedure). The model utilizes a masked self-attention mechanism and consists of multiple stacked decoding blocks. Each stacked decoding block consists of a self-attention layer and fully connected layers. The long-range dependencies of sequences are modeled through a self-attention module. The implementation of the self-attention mechanism and the fully connected layer for each decoding block can be expressed by the following formulas:

(8) $$\mathrm{atten}(Q,K,V)=\mathrm{softmax}(\frac{QK^{T}}{\sqrt{d_{k}}})V,$$

(9) $$\mathrm{hea}d_{i}=\mathrm{atten}(QW_{i}^{Q},KW_{i}^{K},VW_{i}^{V}),$$

(10) $$\mathrm{MultiHead}(Q,K,V)=[\mathrm{hea}d_{1},\cdot \cdot \cdot ,\mathrm{hea}d_{n}]W^{0},$$

(11) $$\mathrm{FNN}(x)=\sigma (xW_{1}+b_{1})W_{2}+b_{2},$$

where *Q* is query, *K* is key and *V* is value vectors, respectively. $d_{k}$ is the dimension of query and key vectors, and *T* is transpose of the matrix. $\mathrm{hea}d_{i}$ is the *i*-th attentional head, [⋅,⋅] is concatenation and all of the *W*'s above are learnable matrices.σ is the activation function GeLU and *b* is bias.

For training, we feed the molecular graph latent vector and the molecular SMILES into the model, and the model is then trained on the next character prediction task so that the latent vector guides the model generation. The generator is trained to maximize the conditional likelihood:

(12) $$\begin{array}{c} L=\text{log}\;p_{\theta }(S_{\mathrm{out}}|V_{\text{latent}},S_{in})\;\\ =\text{log}\;p_{\theta }(s_{1},\;s_{2},\;.\;.\;.\;,\;s_{n}|V_{\text{latent}},S_{in})\;\;\;\;\;\;\\ =\sum_{i=1}^{n}\;\text{log}\;p_{\theta }(s_{i}|s_{1}:s_{i-1},V_{\text{latent}},S_{in}). \end{array}$$

We feed the latent vector into the model, and given a start marker, the model predicts the next marker in the sequence until a stop symbol is generated, thus generating a molecular SMILES.

Since the latent representation of the molecular graph and the input dimension of the decoding block are inconsistent, we add a fully connected layer between the two to map the latent space vector representation to the corresponding dimension vector of the decoding block.

#### 2.2.2 Guided search from predicted inhibition

Although the latent space is already dimensionally reduced relative to the broad drug space, it is still a high-dimensional space. With high-dimensional data, it is difficult to mitigate the impact of dimensional disaster and to continue to explore in the desired direction of growth inhibition in the process of optimization. Inspired by the work of Yang *et al.* (2020), we employ stochastic dominant learning swarm optimization strategy in the latent space. We define a particle as a point in latent space and the fitness as the growth inhibition corresponding to that point. For a seed molecule, the initial population is obtained by sampling around the potential representation, and the population is updated in the direction of minimizing growth inhibition under the guidance of fitness until a point with the minimum global growth inhibition is found. During the update process, points that satisfy the threshold range for growth inhibition are saved and generated (see Supplementary Algorithm S1 for the detailed process). The optimization mainly includes two parts: sampling and updating.

**Sampling.** If the similarity between the sampling point and the seed is too high, the latent space search is inefficient; if the similarity is too low, the sampling point will not be able to keep the characteristics of the seed. We design the following sampling strategy: for a given point, we randomly sample number of populations (NP) (see Supplementary Algorithm S1) Gaussian noises $n_{1},n_{2}\ldots n_{NP-1}∼N(0,D_{1})$. $D_{1}$ is calculated as follows: the encoder is used to obtain the latent representation of the main dataset and compute the variance ${\mathrm{cov}}_{1},{\mathrm{cov}}_{2}\ldots {\mathrm{cov}}_{n}$ for each dimension, where *n* is the dimension of the latent vector. This yields the following:

(13) $$d_{2}=\max({\mathrm{cov}}_{1},{\mathrm{cov}}_{2}\ldots {\mathrm{cov}}_{n}),$$

(14) $$d_{1}=1/2\times d_{2},$$

(15) $$D_{1}=d_{1}\times I,$$

where *I* is an identity matrix and $x_{0}$ represents the latent space representation of the seed. Gaussian noises are then added to the latent space representation vector of the seed:

(16) $$x_{i}=x_{0}+\theta \times n_{j}(j=1,2\ldots NP-1).$$

We set the parameter θ (θ∈[0,1]) to make the noise range controllable. $\left\{x_{0},x_{1},x_{2}\ldots x_{NP-1}\right\}$ form the initial population (see Fig. 1B).

**Updating.** For a given point $x_{i}$, the update is done as follows:

(17) $$\beta =0.5-0.2\times \text{exp}\times (-\frac{f(x_{i})-f(x_{b2})+\xi }{f(x_{i})-f(x_{b1})+\xi }),$$

(18) $$v_{i}=w\times v_{i}+r_{1}\times (x_{b1}-x_{i})+\beta \times r_{2}\times (x_{b2}-x_{i}),$$

(19) $$x_{i+1}=v_{i}+x_{i},$$

where f(g) is a function to calculate individual fitness. We design two f(g) functions: one is a jointly trained FFN predictor, and the other is a Gaussian process (GP) model trained using the training set of the main dataset; both models are used to achieve inhibition consistency. The GP model is a non-parametric model (Williams and Rasmussen, 2006). It is a very lightweight model that takes only a few minutes to train. ξ is a very small number to avoid the denominator being zero. $w,r_{1},r_{2}$ are hyperparameters. It is worth noting that only one fitness function is required in a complete scheme. Designing two and doing experiments is to consider both neural network parametric models (FFN) and non-parametric models (GP), which is an extension of the entire experimental scheme.

### 2.3 Experimental settings

The experimental settings for MDAGS model could be found in Supplementary Material.

## 3 Results

### 3.1 Encoder–predictor joint training analysis

#### 3.1.1 Choice of hyperparameter

In machine learning, especially in the field of deep learning, the choice of hyperparameters has a great impact on the performance of the model. Therefore, in our work, the joint model is also optimized with hyperparameters before jointly training the encoder and predictor. We apply Bayesian optimization (BO) to perform this process (Shahriari *et al.*, 2016). The main idea of BO is that the posterior distribution of a given complex objective function of optimization is computed with a GP. The objective function can have just the input and output specified with unknown internal structure and mathematical properties. The posterior distribution of the objective function is modified until the posterior distribution reasonably fits the real objective distribution so that the current parameters can be better adjusted. In our experiments, the optimized hyperparameters are the number of message-passing steps, neural network hidden size, number of feed-forward layers and dropout probability. We performed 20 optimizations on the training set of the main dataset and used the validation set to select the best hyperparameter combination. The result of hyperparameter optimization is as follows: the number of message-passing steps is 5, the neural network hidden size is 1900, the number of feed-forward layers is 2 and the dropout probability is 0.30000000000000004.

#### 3.1.2 Learned latent space visualization

The generation and optimization of molecules is often concerned with maximizing or minimizing certain properties. In this article, we jointly train an encoder and a predictor to learn the growth-inhibitory property of molecules. In this way, the distribution of molecules in the latent space is organized by growth inhibition.

**Fig. 2.**
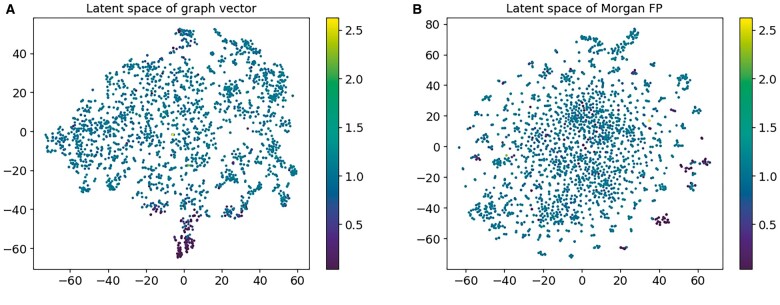
Comparison of the latent space learned by the encoder–predictor and the latent space corresponding to the Morgan fingerprint. (**A**) Learned by the encoder–predictor. (**B**) Calculated using Morgan fingerprints. The value of the color bar represents the strength of antibacterial activity, the stronger the antibacterial activity, the smaller the value

We apply the results of hyperparameter optimization to set up the model and train the model to generate the latent space with the main dataset. We analyzed the latent space’s ability to capture molecular features. Figure 2 shows the mapping of attribute values to latent space attribute values, visualized using t-SNE. From Figure 2A, it can be seen that the molecular representations learned by joint training of the encoder–predictor have a more concentrated distribution for the molecules with obvious growth inhibition, and the molecules with low values are almost all clustered directly below the latent space. To compare with this distribution, we chose the Morgan fingerprint, which is one of the classic fingerprints for feature vectorization of molecules, to compute the fingerprint of the main dataset. We performed the same visualization and found that the calculated molecular latent characterization does not possess the ability to aggregate molecular inhibition (Fig. 2B).

### 3.2 Latent space optimization analysis

#### 3.2.1 Fitness function evaluation

We analyzed the predictive performance of the two designed fitness functions GP and FFN to ensure that the two fitness functions supported the prediction of growth inhibition (Table 1). The experimental growth inhibition *R*^2^ (correlation values) of the two fitness functions were 0.421 and 0.523, the root mean square error (RMSE) were 0.197 and 0.179 and the mean absolute error (MAE) were 0.130 and 0.118, respectively. It can be seen from this table that the performance of the two fitness functions is similar in these indicators, and both have good predictive performance. Therefore, it can be determined that the two fitness functions support the prediction of growth inhibition and can be used for the subsequent potential space-guided search optimization.

**Table 1.** Performance of fitness functions GP and FFN

Fitness function type	MAE	RMSE	*R* ^2^
GP	0.130	0.197	0.421
FFN	0.118	0.179	0.523

#### 3.2.2 Optimization results analysis for selected seeds

In the latent space, we selected four compound molecules from the test set of the main dataset as seeds for optimization: cefazolin sodium, cefotaxime sodium, ceftazidime and minocycline hydrochloride. The first three of these four compounds are cephalosporin antibiotics and the fourth is a tetracycline antibiotic. Among them, Compounds 1 and 3 have broad-spectrum antibacterial ability and Compounds 2 and 4 are active against gram-positive and gram-negative bacteria.

For the four selected seed compounds, predicted growth inhibition was calculated with GP and FFN and compared with the actual growth inhibition (Supplementary Table S2). The prediction results of the two functions are slightly different. In addition, we counted the number of potential threshold range representations corresponding to the four seeds generated by the GP and FFN fitness function optimization (Supplementary Table S3). The FFN fitness function has more latent space representations than the GP optimization. We believe that this is related not only to setting different thresholds but also to the prediction accuracy of the two fitness functions. Exploring more successful latent space representations may mean exploring a wider space. Supplementary Figure S2 verifies our conjecture. The latent representations optimized with the GP fitness function are more evenly spread around the seeds, but the latent representations optimized with the FFN fitness function have been further explored in a certain direction.

Our goal is to find new molecules with stronger growth inhibition. We plotted the inhibition values corresponding to the main dataset and the latent representations optimized by the GP and FFN fitness functions (Supplementary Fig. S3). It can be seen from the figure that the predicted inhibition value of the latent representation obtained by the optimization of the two fitness functions is significantly lower than that of the main dataset.

### 3.3 Generation analysis

#### 3.3.1 Generation performance comparison

It is important for the generative model to generate chemically valuable, novel and unique molecules. This ensures that the generator has the ability to broadly explore new spaces beyond the existing chemical space without overfitting existing data. Therefore, we evaluate the generative ability of the model on multiple metrics and compare it with two benchmarks—MOSES and GuacaMol. It is worth noting that although we use the MolGPT framework in this article, our generation idea and method differ from the original. We believe these have an impact on the generative power of the model. Therefore, we also compared with the MolGPT model.

On the MOSES benchmark, we compare our model with five benchmarks, CharRNN, AAE, VAE, JTN-VAE and LatentGAN, in addition to the MolGPT model (Table 2). In addition to validity, novelty and uniqueness, evaluation metrics include filters and internal diversity. Filters are mainly used to evaluate the proportion of filtered generated molecules applied during dataset construction. The resulting molecules, while chemically valid, may contain unnecessary fragments. Therefore, when building the MOSES training dataset, molecules with these fragments were removed, and the model is expected to avoid producing them (Polykovskiy *et al.*, 2020). Internal diversity assesses the chemical diversity within the generated set of molecules to avoid model collapse (models tend to produce several fixed types of data) (Benhenda, 2017). The results showed that our model significantly outperformed the other models on the novelty metric, suggesting that the model can generate new molecules that are completely different from the training set. In addition, it also has comparable performance to the baseline model on the other four metrics.

**Table 2.** Performance comparison on the MOSES benchmark

Model	Validity (↑)	Uniqueness@10k (↑)	Novelty (↑)	Filters (↑)	IntDiv (↑)
CharRNN	0.9748	**0.9994**	0.8419	0.9943	0.8562
AAE	0.9368	0.9973	0.7931	0.9960	0.8557
VAE	0.9767	0.9984	0.6949	0.9970	**0.8558**
JTN-VAE	**1.0000**	0.9996	0.9143	0.9760	0.8551
LatentGAN	0.8966	0.9968	0.9498	0.9735	0.8565
MolGPT	0.9900	0.9986	0.8093	**0.9972**	0.8526
MDAGS	0.9556	0.9984	**0.9925**	0.9948	0.8534

The bold value in each column corresponds to the optimal value in all comparison models under this indicator.

On the GuacaMol benchmark, we compare our model with four benchmarks, Graph MCTS, AAE, ORGAN and VAE, in addition to the MolGPT model (Table 3). In addition to validity, novelty and uniqueness, the evaluation metrics also include KL divergence and FCD metrics. KL divergence measures how well one probability distribution approximates another (Kullback and Leibler, 1951). This metric reflects the ability of the model to fit the distribution of the data. FCD is a measure of how close the distribution of the generated data is to the distribution of molecules in the training set (Preuer *et al.*, 2018). This metric reflects the model’s ability to incorporate important chemical and biological features. The results show that our model has the best performance on uniqueness, KL divergence and FCD. This shows that the model can generate novel new molecules while fitting the distribution of the dataset.

**Table 3.** Performance comparison on the GuacaMol benchmark

Model	Graph MCTS	AAE	ORGAN	VAE	MolGPT	MDAGS
Validity	**1.000**	0.822	0.379	0.870	0.970	0.929
Uniqueness	**1.000**	**1.000**	0.841	0.999	0.998	**1.000**
Novelty	0.994	0.998	0.687	0.974	**1.000**	0.984
KLD	0.522	0.886	0.267	**0.982**	0.981	**0.982**
FCD	0.015	0.529	0.000	0.863	0.736	**0.874**

The bold value in each row corresponds to the optimal value in all comparison models under this indicator.

#### 3.3.2 The impact of transfer learning

For language generation models, it is necessary to train on a large dataset to learn grammar rules. Considering the small number of main datasets, direct training cannot learn SMILES grammar rules well, which affects the generation effect. We chose to learn grammar rules on two large datasets, MOSES and GuacaMol, and then use the main dataset for fine-tuning to generate antibiotic-specific compounds.

We investigated whether generators benefit from transfer learning. We plotted the loss curves of the model trained directly and the models fine-tuned after pretraining on the MOSES and GuacaMol datasets, and then evaluated each model’s ability to fit the data (Supplementary Fig. S4). In addition, the test set of the main dataset was generated with three trained generators after removing the duplicate data in the MOSES and GuacaMol datasets, and the ability of the generators to regenerate was evaluated (Supplementary Table S4). The results show that the model trained directly on the master dataset performs less well than the pretrained and fine-tuned models. Additionally, we found that the model pretrained and fine-tuned on the GuacaMol dataset outperformed its MOSES dataset counterpart. We speculate that this may because the GuacaMol dataset has a more similar distribution to the main dataset. Supplementary Figure S5 verifies our estimate. Considering the excellent performance of the model on the GuacaMol-pretrained dataset, we use this model in subsequent generation tasks.

### 3.4 Generated results verification

#### 3.4.1 Generative spaces analysis

We generated 7496 and 11 924 chemically usable molecules with the trained generators for GP and FFN, respectively, as successful latent representations of fitness optimization. We explored the spatial intersection generated by the two methods (Supplementary Fig. S6a); there were 476 repeated molecules explored in the two methods, and 7020 and 11 448 different molecules were generated by each exploration, respectively. The two spanning spaces are more intuitively displayed using TMAP (Supplementary Fig. S6b), which calculates the similarity based on ECFP fingerprints to construct a minimum spanning tree. The tree is drawn and displayed by Fearun, and a point in the figure represents a molecule (Probst and Reymond, 2018). Since the two modalities explore different chemical spaces, further analysis of both chemical spaces is warranted.

**Fig. 3.**
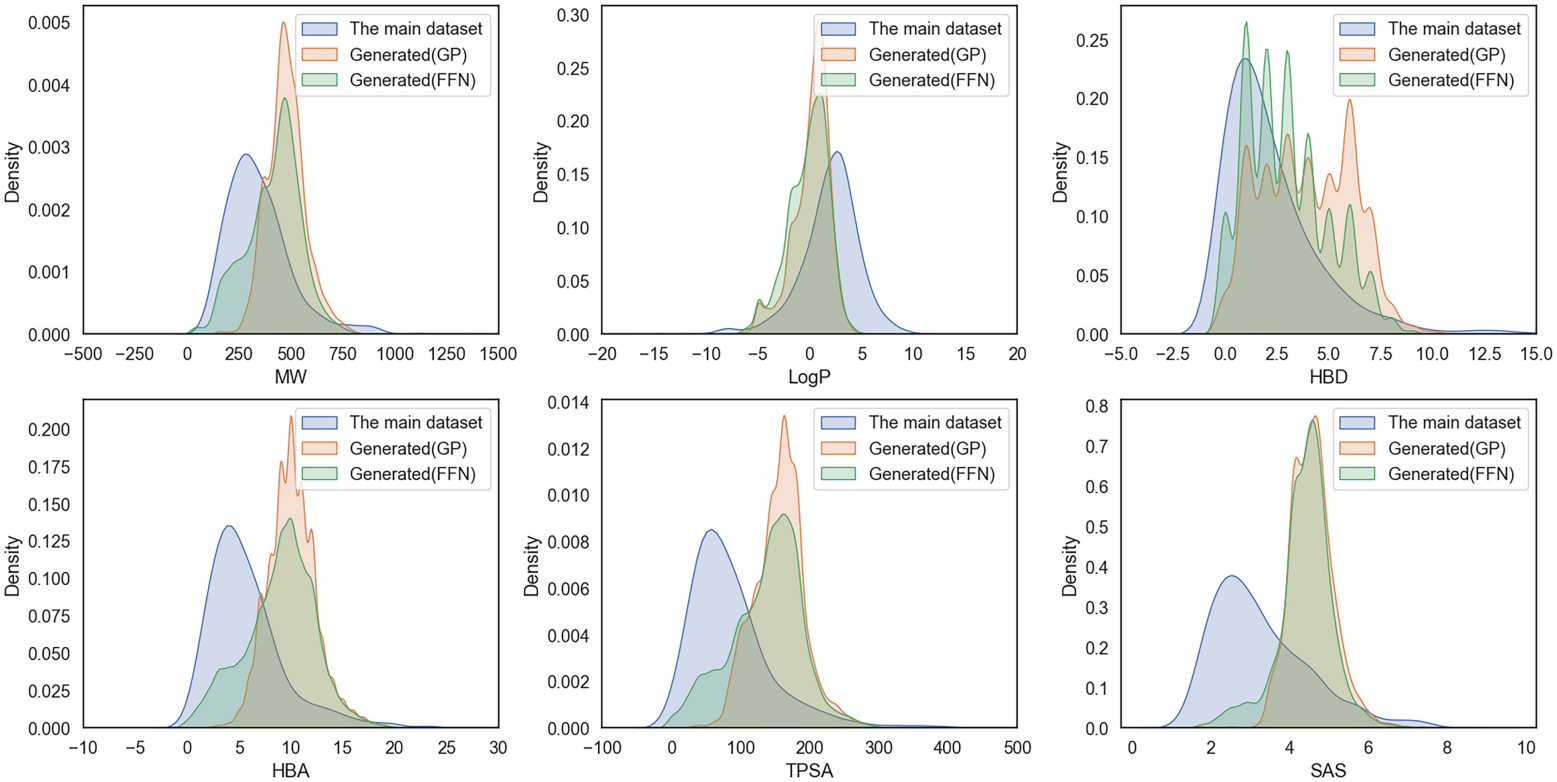
Probability distributions of molecular properties for the two generated spaces compared to the main dataset. Calculated property distributions: MW, LogP, HBD, HBA, TPSA and SAS. Lines represent kernel density estimates for individual attributes of the main dataset and the two generated datasets

We further explored the property distribution of the molecules in the two generative spaces with the main dataset (Fig. 3). The distribution of the two generated datasets is almost the same as that of the main datasets of several important attributes [MW, LogP, number of hydrogen bond donors (HBD), number of hydrogen bond acceptors (HBA), topological polar surface area (TPSA) and synthetic accessibility (SAS)] on the whole, but the individual properties are slightly different: for MW, LogP and HBD, the generated molecules are consistent with the main datasets. However, half of the generated molecules do not conform to the principle of HBA < 10 in the five principles of drug like. And the TPSA of the resulting molecule is more difficult to penetrate the cell membrane than the dataset. The SAS distribution of the generated molecules is more like 10, which means that the generated molecules are relatively difficult to synthesize compared to the dataset. For the performance on HBA, TPSA and SAS, we considered that the generated molecules were affected by the properties of the corresponding seeds.

#### 3.4.2 Generated potent antibiotic verification

**Fig. 4.**
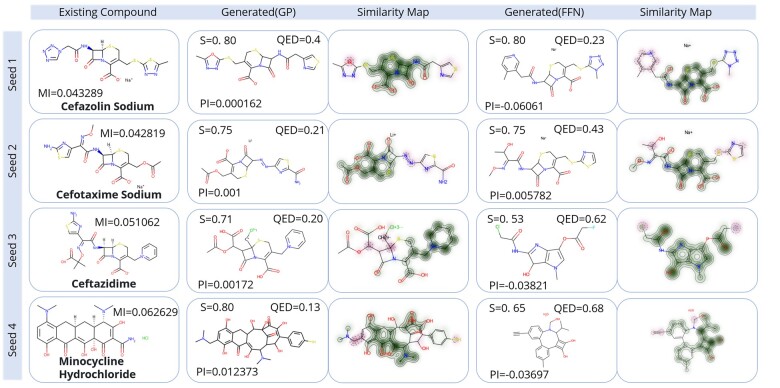
Sample results for potent antibiotic generation. Each row corresponds to the result of a seed. The molecules in the first column are the seeds, and MI is the Mean_Inhibition value of the experiment. The molecule in the second column is the molecule obtained by the GP fitness function and the molecule in the fourth column is the molecule obtained by the FFN fitness function. *S* is the Tanimoto similarity between the molecule and the corresponding seed, QED is the drug-like property of the molecule and PI is the predicted inhibition value. The third and fifth columns are the similarity maps between the molecules in the second and fourth columns and the corresponding seeds. The darker the color is, the more similar the substructure of the molecule and the seed is. The first column in bold is the name of each antibiotic molecule

In this section, we further verify the properties of the generated molecules. For the two generative spaces, we removed the molecules with a similarity >0.80 from the generation results of each seed and then sorted the molecules in the order of Tanimoto similarity from high to low and inhibition value from small to large. The compounds at the resulting first position were further analyzed (Columns 2 and 4 of Fig. 4). These eight compounds obtained stronger growth inhibition ability (lower PI value) under the condition of ensuring higher similarity with seeds. In other words, these molecules have the potential to have stronger antibacterial activity while their other properties remain similar to those of existing antibiotics. The third and fifth columns of Figure 4 show the similarity maps of the eight molecules and the corresponding seeds so that similar substructures between them can be visually observed. The SMILES corresponding to the seeds in Figure 4 and the eight sampled seeds are shown in Supplementary Table S5. We also calculated several properties of the seed molecule and the eight sampled compounds—MW, Log P, HBD, HBA, TPSA and SAS (Supplementary Table S6)—and found that the eight sampled molecules and the corresponding seed molecules have very similar properties.

In addition, we queried the top three nearest neighbors of these eight compounds in DrugBank (Supplementary Fig. S7). The first three nearest neighbors of these eight compounds are almost all antibiotics, and the functions of the three nearest neighbors are almost the same as the corresponding seed of the compound [e.g. the first compound was optimized by the first seed (cefazolin, a cephalosporin antibiotic), and its three nearest neighbors are also cephalosporin antibiotics, which is consistent with the antibiotic function in the seeds]. This further demonstrates the bacteriostatic potential of these eight sampled molecules.

**Fig. 5.**
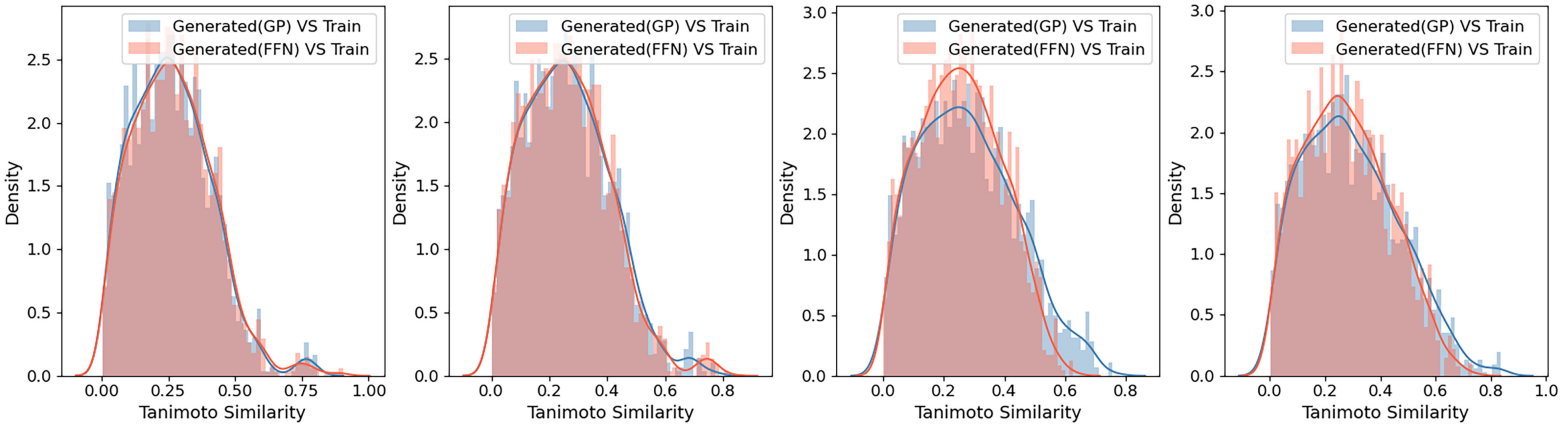
Tanimoto similarity of the eight sample compounds to the training set of the main dataset. For each seed, histograms show the similarity of molecules sampled from the chemical space generated by GP or FFN fitness optimization to the training set of the main dataset, and lines represent the kernel density estimates for the respective histograms

We also calculated the Tanimoto similarity between these eight compounds and the training set of the main dataset (Fig. 5). We found that the similarities between the generated molecules and the dataset have a consistent distribution, and the similarities are relatively low, almost all closer to 0 rather than 1, indicating that these molecules are not simply replicating the dataset.

#### 3.4.3 Molecular visualization generated along the optimized direction

The goal of latent space optimization is to make the population move in the direction of stronger inhibition under the guidance of fitness to explore compounds with stronger antibacterial activity. We sampled the molecules generated by latent space exploration in sequential equal steps and observed the similarity of the sampled molecules to the corresponding seeds and their inhibitory properties (Supplementary Figs S8 and S9). We found that the population is always able to continue exploring in a more inhibitory direction while guaranteeing a higher similarity to the seed. The corresponding SMILES of the compounds in Supplementary Figs S8 and S9 are shown in Supplementary Tables S7 and S8, respectively.

## 4 Conclusion

In this article, we report on MDAGS, a generation method of potent antibiotic design. The method incorporates two novel ideas: the encoder and predictor are jointly trained to learn a latent property space, and an attribute-guided optimization strategy is employed in the latent space to enable the model to explore in the direction of the expected properties of the molecule. Doing so aggregates molecules with similar properties, thereby reducing exploration of the vast space and avoiding exploration in directions that may not make sense. In addition, considering that the generator does not contribute to the learning of the latent space, we decouple the encoder and generator, which greatly reduces the model complexity.

Our findings suggest that MDAGS can serve as a novel and practical method for effective antibiotic design. Visualization of the designed latent space shows that the joint training of the model captures molecular properties so that molecules cluster in spatially distinct locations according to antibacterial activity. Validation of the two fitness functions demonstrates the ability of the model to predict the antibacterial activity of the underlying representation, thereby supporting the optimization of molecules in the potential property space. On the benchmark task of unlabeled molecule generation, MDAGS shows comparable or even better performance than the benchmark model. Furthermore, in the antibiotic molecule generation task, the analysis of the generated molecules shows that our method is able to generate molecules with other properties comparable to existing antibiotics but with better antibacterial activity, and there is a large difference between these generated molecules and existing real data. Finally, the equal-step sampling of the generated results provides insight into how efficiently the method can navigate the property space to find improved molecules with desired properties.

Given the generality of MDAGS, we believe it will impact other drug discovery cases. For example, designing a more inhibitory antitumor drug requires little to no model changes, just data replacement. However, the method also has limitations. In this work, we only focus on and optimize one property, i.e. antibacterial activity; however, when a drug acts on an organism, many factors need to be considered, such as low toxicity and better pharmacokinetic properties. Our current method does not take these factors into account.

Overall, our work provides a new approach for the generation and optimization of molecules, especially potent antibiotic compounds. This is undoubtedly of great significance for addressing today’s increasingly serious problem of antibiotic resistance. At the same time, we believe our work will positively impact other drug discovery cases. In future work, we will further explore optimizing our method to consider multi-objective optimization to improve the success rate of compounds in clinical trials.

## Supplementary Material

btad059_Supplementary_Data

## Data Availability

The Drugs and Natural Products data underlying this article are avaliable in https://github.com/LiangYu-Xidian/MDAGS. The MOSES data underlying this article are avaliable in https://github.com/molecularsets/moses. The GuacaMol data underlying this article are avaliable in https://github.com/BenevolentAI/guacamol.
