# Supplementary material for: Potent antibiotic design via guided search from antibacterial activity evaluations
Source: Bioinformatics. 2023 Jan 27;39(2):btad059. doi: 10.1093/bioinformatics/btad059 (PMC9897189; doi:10.1093/bioinformatics/btad059)
Supplement: btad059_Supplementary_Data [file btad059_supplementary_data.docx]

**Supplementary Materials**

## Supplementary algorithm

Algorithm 1 describes the SDLSO optimizer used in our implementation.

| **Algorithm 1** Molecule Optimization based on SDLSO |
| --- |
| **input**: $NP$(swarm size), $FES$(maximum fitness evaluations), $w,r_{1}$ and $r_{2}$(update individual weights), $\dim$(dim of graph vector), $mean$(mean of sampling Gaussian noises), $\mathrm{cov}$(covariance of sampling Gaussian noises), $theta$(Similarity between sampled Gaussian noises and seed), $fit\_\min$ and $fit\_\max$(inhibitory interval for particles to be saved), $seeds$(encoded vector of seeds molecule graph)  1: for $i$ in range ($len$($seeds$)):  2: $seed$=$seeds_{i}$;  3: sample $NP-1$ Gaussian noises ;  4: initialize $x_{0}=seed$,, random initialize$v_{0},v_{1}\ldots v_{NP-1}$;  5: calculate fitness(inhibition) of $x$ according to GP or FFN;  6: $fit\_\max=fit_{0}$;  7: for $j$ in range ($NP$):  8: if $fit_{j}<=fit\_\max$ && $fit_{j}>=fit\_\min$:  9: save $x_{j}$ and $fit_{j}$;  10: $fes=NP$;  11: while ($fes<NP$):  12: for $k=1:NP$:  13: select two random particles from the swarm: $b1$,$b2$;  14: if $fit_{b1}<=fit_{k}$ && $fit_{b2}<=fit_{k}$:  15: if $fit_{b2}<fit_{b1}$:  16: swap $b1$ and $b2$;  17: update particle $x_{k}$;  18: calculate the fitness(inhibition) of $x_{k}$ according to GP or FFN;  19: if $fit_{k}<=fit\_\max$ && $fit_{k}>=fit\_\min$:  20: save $x_{k}$ and $fit_{k}$;  21: $fes++$;  22: obtain the global best solution $gbest$ and its fitness(inhibition) $fit_{gbest}$; |
| **output**: saved $x$ and $fit$; |

## Experimental settings

**Datasets.** We randomly divided the main dataset into a training set, validation set and test set at a ratio of 8:1:1 (seed 0). For the MOSES dataset and the GuacaMol dataset, we keep the original divisions.

**Encoder-predictor.** The model uses the PyTorch framework and sets the seed to 0. The number of iterations is set to 30, the number of warmup epochs is set to 2, the initial learning rate is set to 1e-4, the final learning rate is set to 1e-4, and the maximum learning rate is set to 1e-3. The learning rate linearly increases from the initial learning rate to the maximum learning rate between 0 and the number of warmup epochs and exponentially decreases from the maximum learning rate to the final learning rate between the number of warmup epochs and the number of iterations. The batch size is set to 50 and num_workers is set to 8. The optimizer is the Adam optimizer, with $\beta_{1}$ set to 0.9 and $\beta_{2}$ set to 0.999. For the message aggregation of the encoder’s directed message passing mechanism, the mean aggregation method is used. The activation function of the predictor uses the ReLU activation function.

**Latent Space Optimization.** We set $seed$ to 0, $w$ to 0.8, $r_{1}$ and $r_{2}$ to 0.5, $NP$ =600, $FES$ =12000, and $theta$ =0.5. For $fit\_\max$ , we set the two fitness functions GP and FFN to the respective predicted inhibition values of the compound seeds. Considering the prediction error of GP and FFN, we think it is unreasonable to set the same $fit\_min$. Therefore for $fit\_min$ , GP fitness function is set to 0 and FFN fitness function is set to -0.3, which is to predict the test set of the main data set by GP and FFN respectively and find the minimum value. In the experiments, we sequentially seeded four compounds and optimized them.

**Generator.** The generator uses the PyTorch framework. In the experiment, we set the number of stacked decoding blocks to 8, the output dimension of the self-attention layer to 256. The fully connected layer has three layers: input layer, hidden layer and output layer. The input dimension of the fully connected layer to 256, the hidden layer to 1024, and the output layer dimension to 256. The activation function to set to the GeLU function. The number of iterations of the model is set to 10, the batch size is set to 512, the learning rate is set to 10e-4, and the optimizer is set to the Adam method, where $\beta_{1}$ is set to 0.9 and $\beta_{2}$ is set to 0.999. The starting token of the generator is set to c, which is the most frequent letter in the first letter position in the test set of the main dataset.

**Other settings.** Model training is conducted on the CentOS Linux release 7.7.1908(Core) system. The CPU is an Intel(R) Xeon(R) Gold 6230 CPU @2.10GHz with the memory of 188 GB. The GPU is a NVIDIA GeForce RTX 3080 with the memory of 42 GB. The model is trained on the GPU and the training process took approximately 190 hours. The code language is Python 3.6, and the model is built using PyTorch 1.10.1. Other packages and configurations are as follows: Fcd-torch=1.0.7, Matplotlib=3.3.4, Numpy=1.19.2, Pandas=1.5.1, Pip=0.40.0, Rdkit=2021.03.4, Scikit-learn=0.24.2, Scipy=1.5.4, Seaborn=0.11.2, Torchaudio=0.10.1, Torchvision=0.11.1, Tqdm=4.63.0.

## Supplementary figures

**
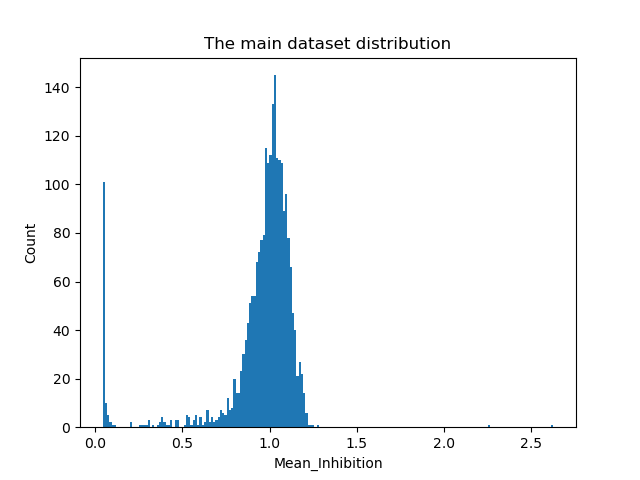
**

**Fig. S1. Distribution of growth inhibition in the main dataset.** The abscissa is the value of growth inhibition, and the ordinate is the number of corresponding molecules**.**

**
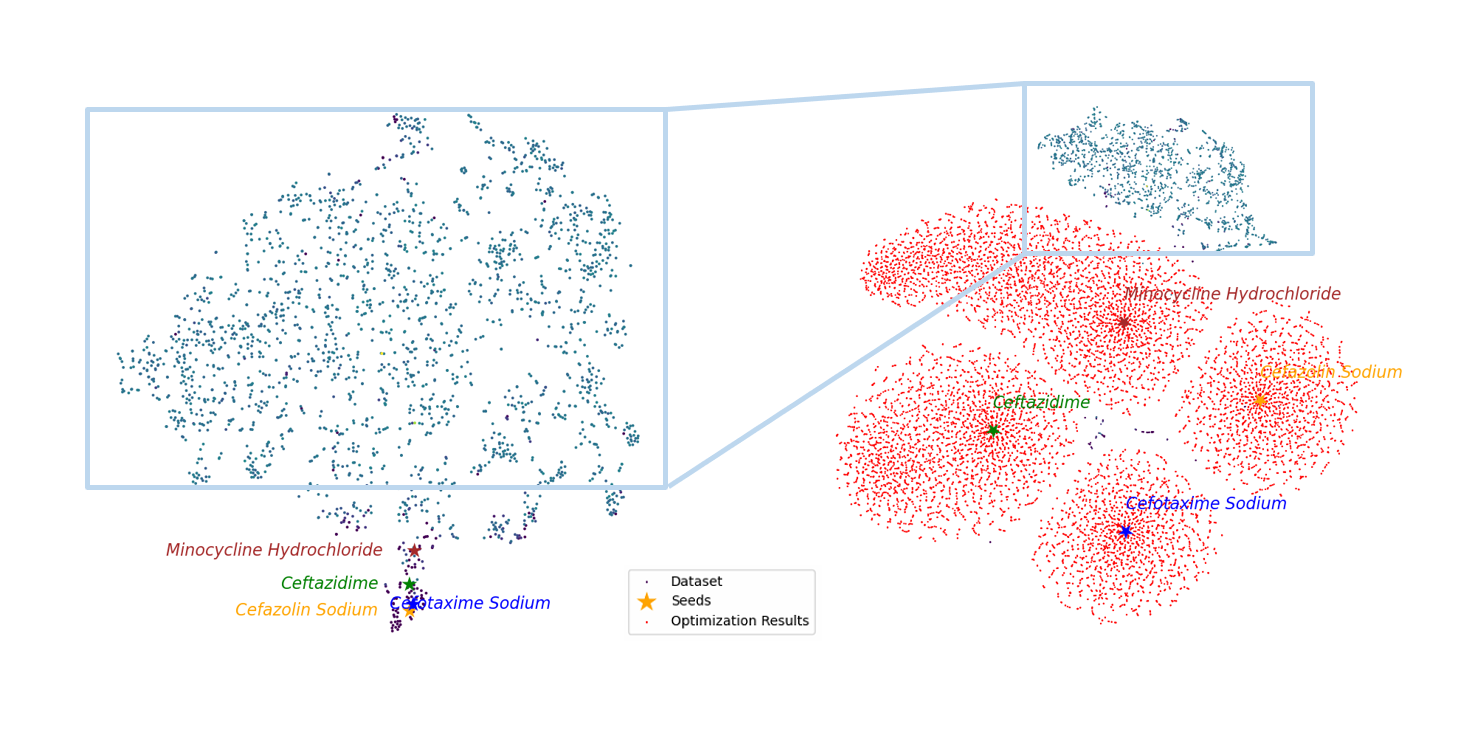
**

(a)


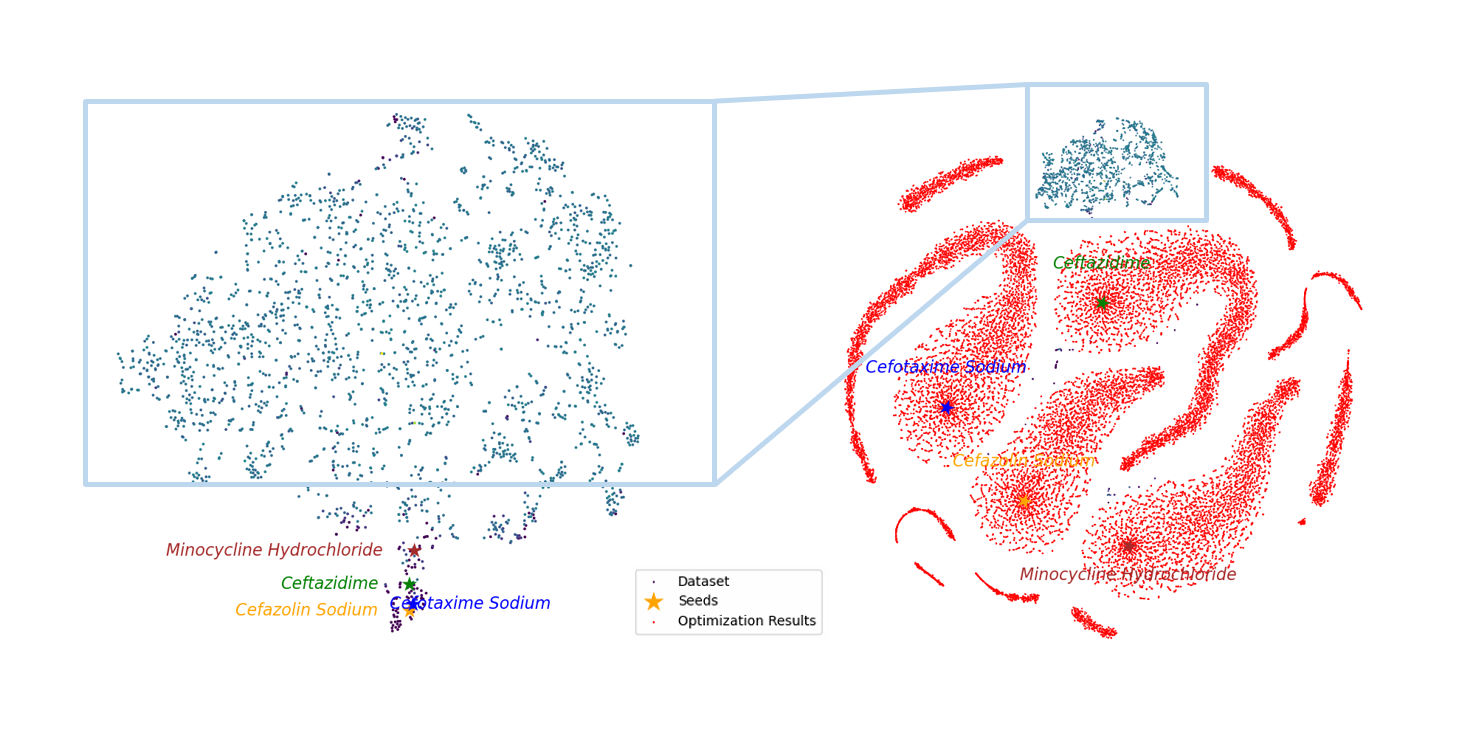


(b)

**Fig. S2. Comparison of original space and optimized space.** (a) comparison of the original latent space and the optimized latent space (GP). (b) comparison of the original latent space and the optimized latent space (FFN). The left and right images in (a) and (b) are the original latent space and the optimized latent space, respectively. Pentagrams mark the positions of seed compounds before and after optimization. The light blue boxes are the corresponding positions in the latent space of some main datasets before and after optimization.


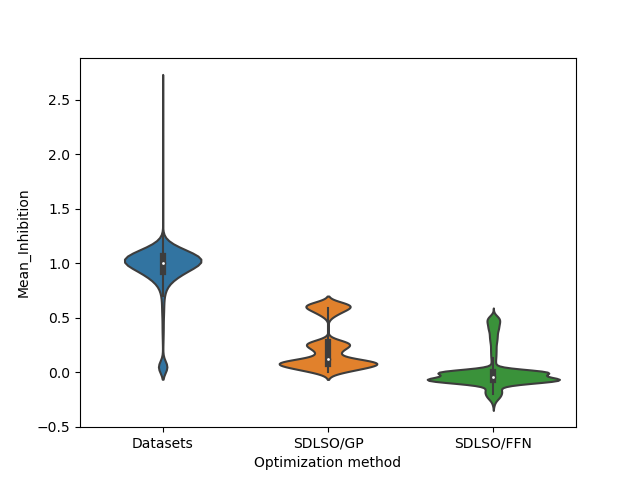


**Fig. S3. Comparison of growth inhibition of the dataset and points obtained by optimization.** The abscissa represents the data type: the main dataset, GP and FFN. The GP and FFN data are the latent representations obtained by the fitness function optimization. The ordinate represents either the experimental growth inhibition (main dataset) or the predicted growth inhibition (GP and FFN, respectively).


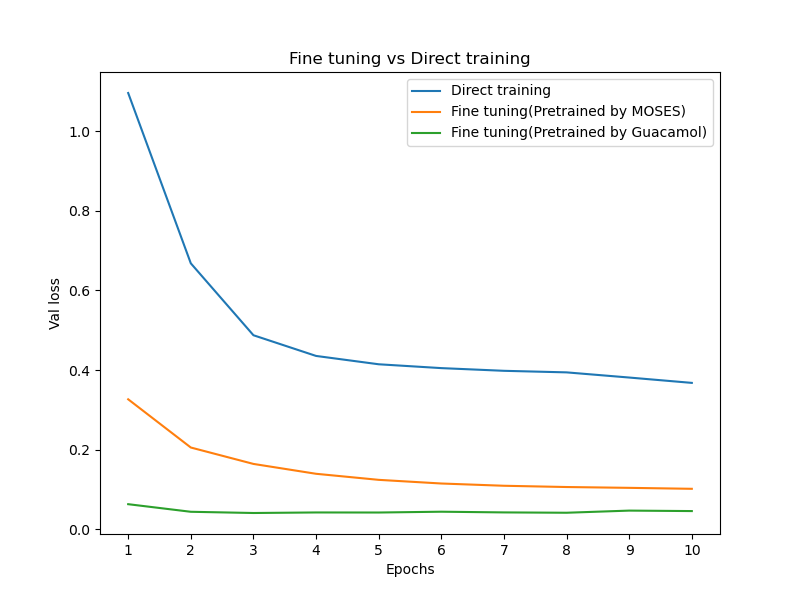


**Fig. S4. Direct training loss comparison with fine-tuned model after training.** The blue line in the figure is the loss of training directly on the main dataset, the yellow line is the loss during fine-tuning after pre-training on the MOSES dataset, and the green line is the loss of fine-tuning process after pre-training on the GuacaMol dataset. The fine-tuned dataset is the main dataset.


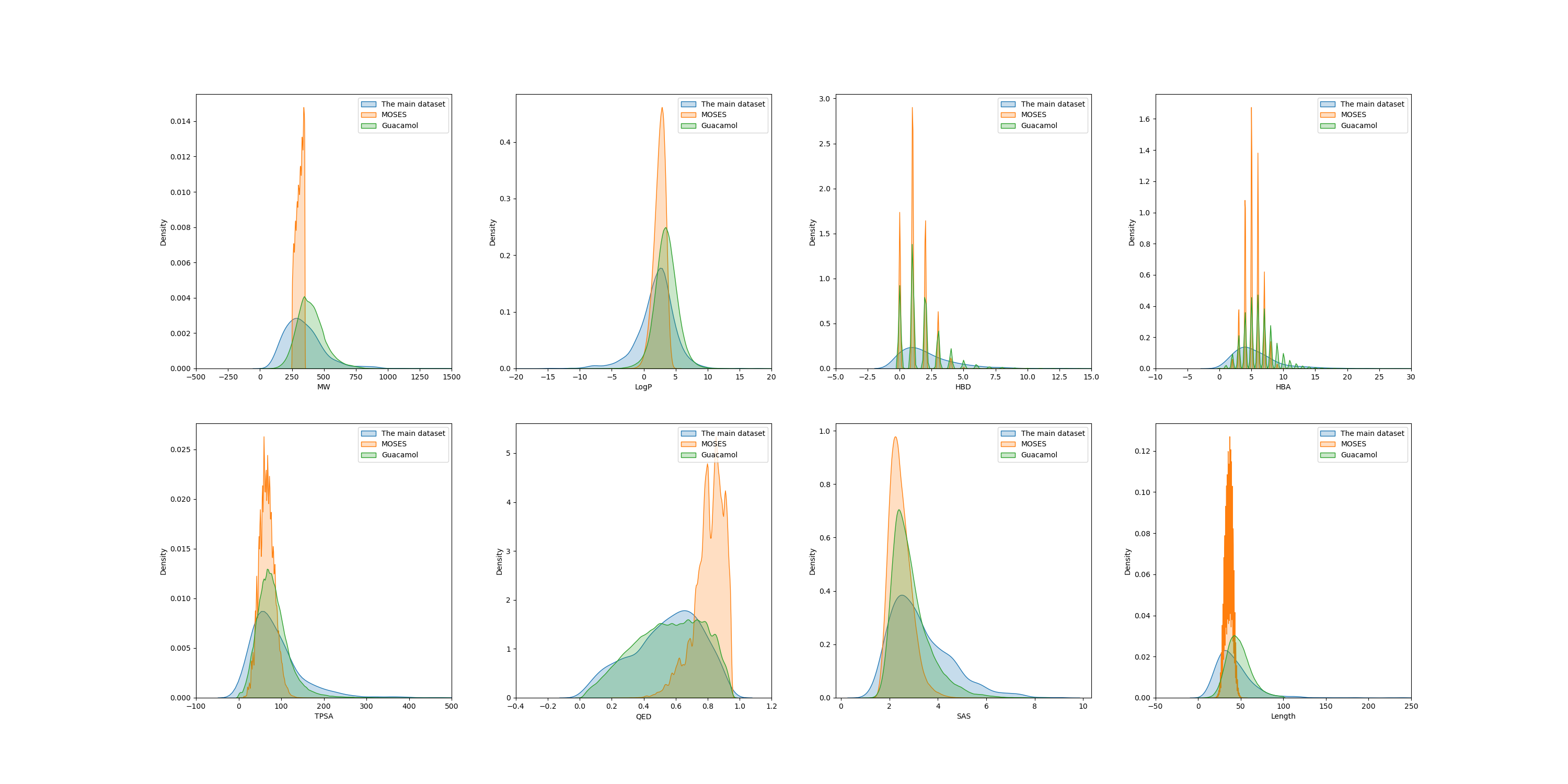


**Fig. S5. Probability distributions of properties of molecules in the main dataset, MOSES and GuacaMol datasets.** Distribution of calculated properties: molecular weight (MW), LogP, number of hydrogen bond donors (HBD), number of hydrogen bond acceptors (HBA), topological polar surface area (TPSA), drug-likeness (QED), synthetic accessibility (SAS), SMILES length (Length). Blue or orange or green lines represent kernel density estimates for individual properties.

**
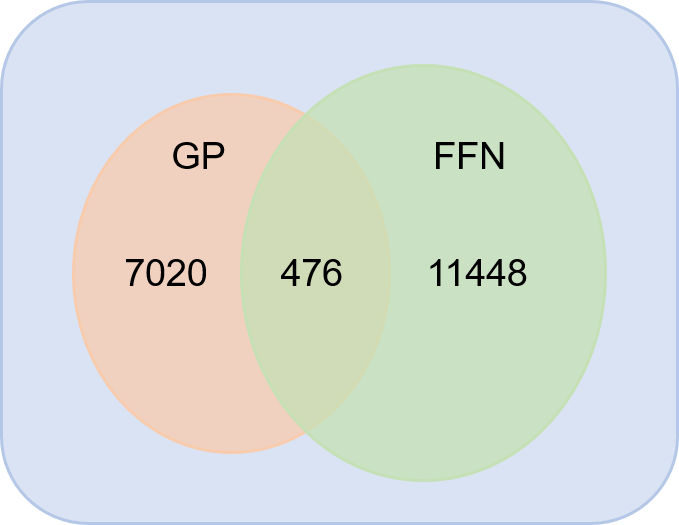

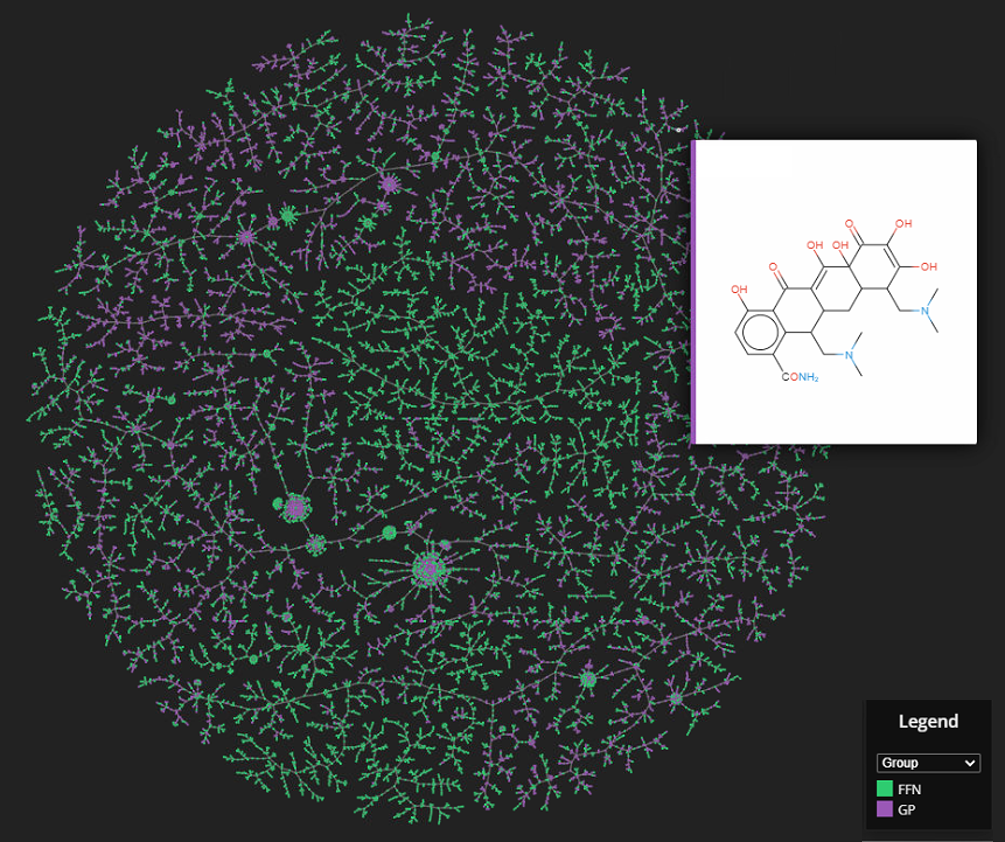
**

(a) (b)

**Fig. S6. Generate space visualization.** (a) and (b) are both analyses of the chemical space generated by optimization of GP and FFN as fitness functions, respectively. (a) generate space intersections. (b) visualization of generate space.It is drawn and displayed by TMAP and Fearun, and a point in the figure represents a molecule.


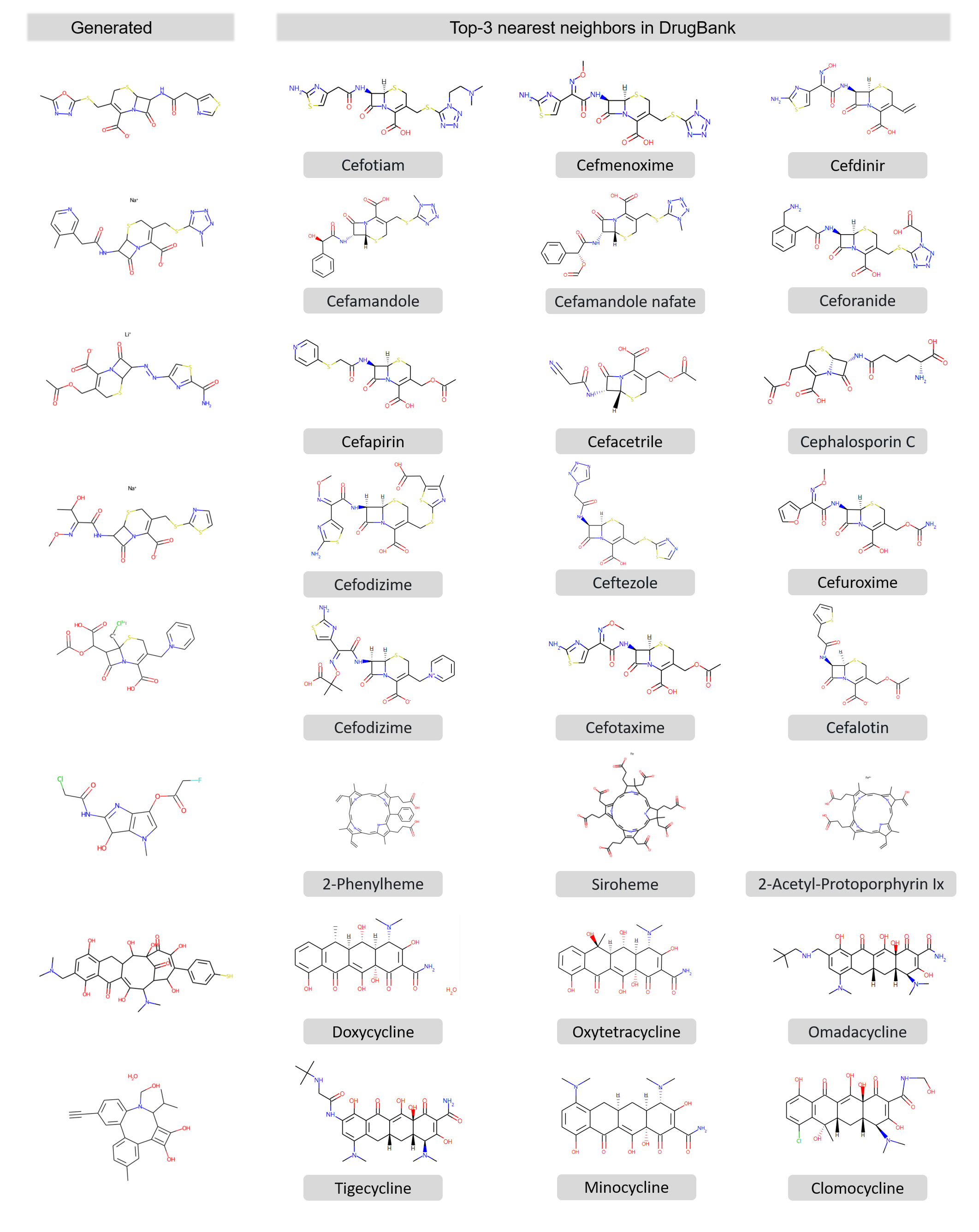


**Fig. S7. The top three nearest neighbors of the 8 sample compounds in DrugBank.** The 8 molecules in the leftmost column are sampled molecules. The three columns on the right are the first three compound molecules that are most similar to the corresponding sampled molecules in DrugBank (arranged from left to right in descending order of similarity), and the gray box below each compound molecule is its name.


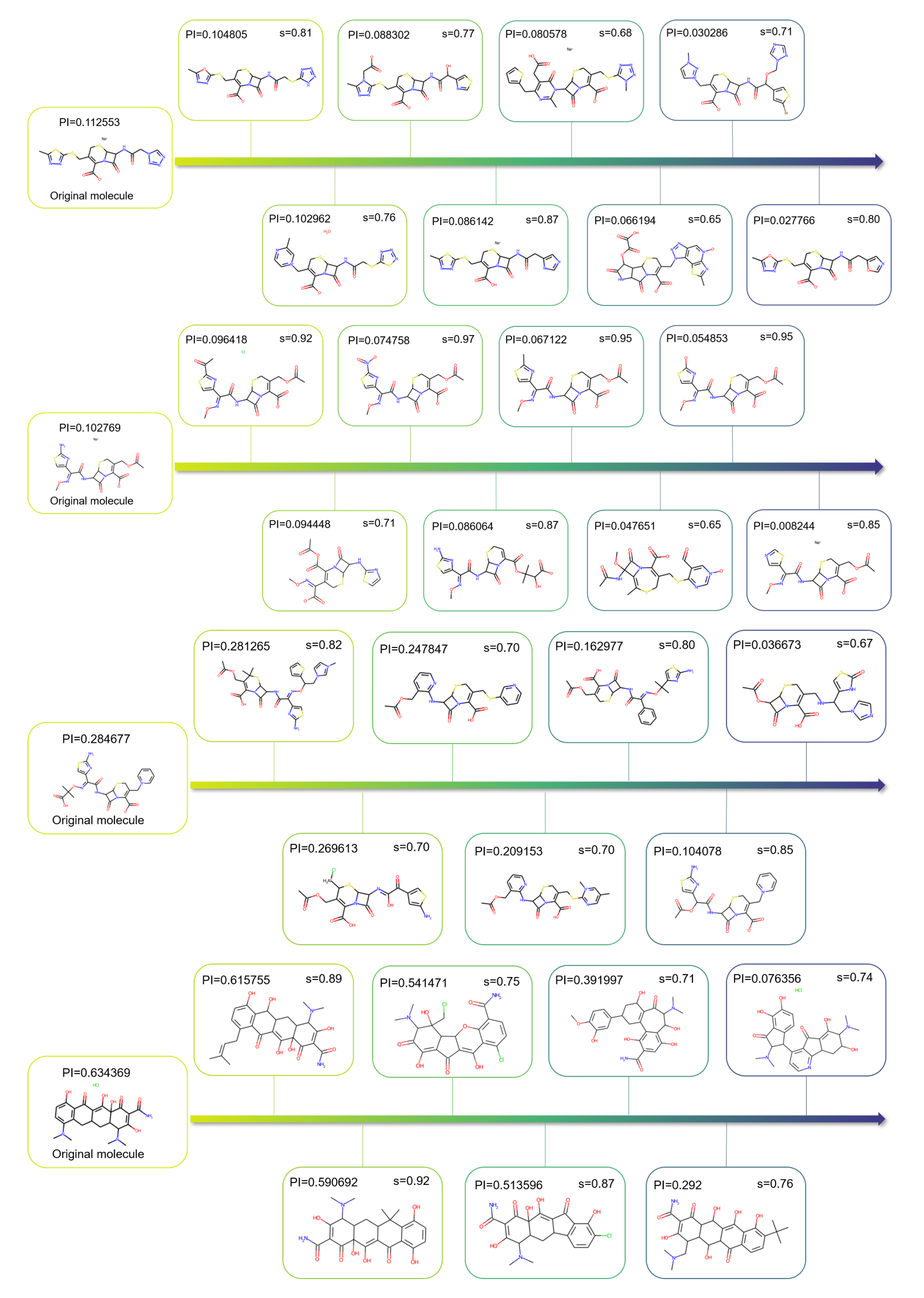


**Fig. S8. Molecular generated along the optimized direction (GP).** For 4 seeds, the molecules generated along the optimized path with GP as fitness are sampled with equal step size, where PI is the predicted growth inhibition value and s is the similarity of the compound to the seed.


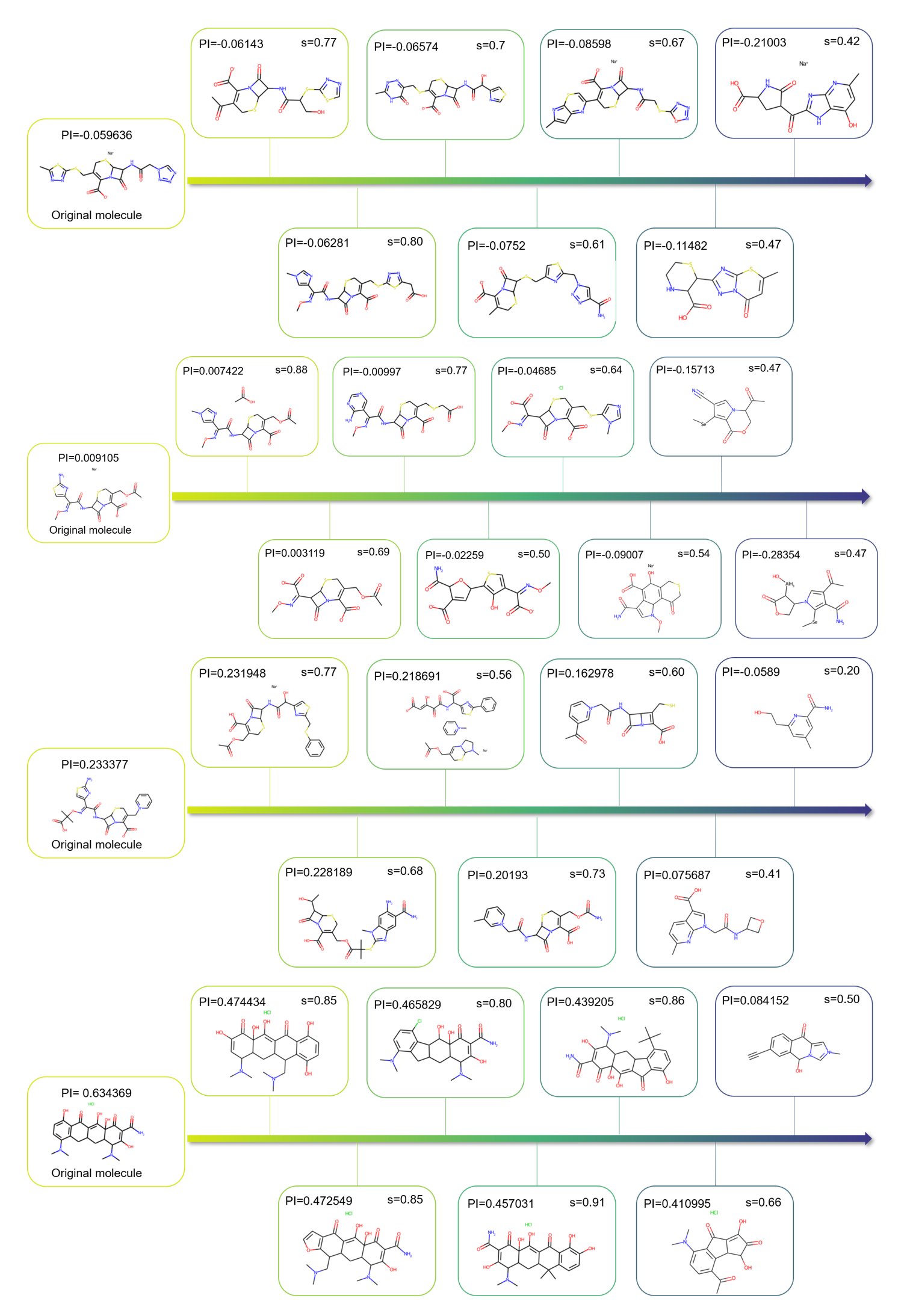


**Fig. S9. Molecular analysis generated along the optimized direction (FFN).** For 4 seeds, the molecules generated along the optimized path with FFN as fitness are sampled with equal step size, where PI is the predicted growth inhibition value and s is the similarity of the compound to the seed.

## Supplementary tables

**Table S1.** Atom Features and Bond Features.

| Feature type | Features |
| --- | --- |
| atom | atomic number |
|  | number of bonds for each atom |
|  | formal charge(integer electronic charge assigned to atom) |
|  | chirality(unspecified, tetrahedral CW/CCW, or other) |
|  | number of bonded hydrogens |
|  | hybridization(sp, sp2, sp3, sp3d, or sp3d2) |
|  | aromaticity(whether this atom is part of an aromatic system) |
|  | atomic mass(divided by 100) |
| edge | bond type (single/double/triple/aromatic) |
|  | conjugation(true or false) |
|  | ring membership(true or false) |
|  | stereochemistry(none, any, E/Z or cis/trans) |

**Table S2.** Inhibition for the four selected compounds.

| Number | Compound | Experimental Inhibition | Predicted Inhibition/GP | Predicted Inhibition/FFN |
| --- | --- | --- | --- | --- |
| Seed 1 | Cefazolin Sodium | 0.043289 | 0.112553 | -0.059636 |
| Seed 2 | Cefotaxime Sodium | 0.042819 | 0.102769 | 0.009150 |
| Seed 3 | Ceftazidime | 0.051062 | 0.284677 | 0.233377 |
| Seed 4 | Minocycline Hydrochloride | 0.062629 | 0.634369 | 0.476532 |

**Table S3.** Counting Seed Molecules Optimization Results.

| Seed | Count/GP | Count/FFN |
| --- | --- | --- |
| Seed 1 | 2600 | 5572 |
| Seed 2 | 2439 | 6970 |
| Seed 3 | 3875 | 7327 |
| Seed 4 | 5092 | 7518 |
| All | 14006 | 27450 |

**Table S4.** Generator reproduction performance.

| Model | Direct training | Fine tuning (MOSES) | Fine tuning (GuacaMol) |
| --- | --- | --- | --- |
| Reprod | 0% | 8.70% | 13.91% |

**Table S5.** SMILES for 4 seeds and 8 sample compounds.

| Compound | Type | SMILES |
| --- | --- | --- |
| Cefazolin Sodium | Original | Cc1nnc(SCC2=C(C(=O)[O-])N3C(=O)C(NC(=O)Cn4cnnn4)C3SC2)s1.[Na+] |
|  | Optimized (GP) | Cc1nnc(SCC2=C(C(=O)[O-])N3C(=O)C(NC(=O)Cc4cscn4)C3SC2)o1 |
|  | Optimized (FFN) | Cc1ccncc1CC(=O)NC1C(=O)N2C(C(=O)[O-])=C(CSc3nnnn3C)CSC12.[Na+] |
| Cefotaxime Sodium | Original | CON=C(C(=O)NC1C(=O)N2C(C(=O)[O-])=C(COC(C)=O)CSC12)c1csc(N)n1.[Na+] |
|  | Optimized (GP) | CC(=O)OCC1=C(C(=O)[O-])N2C(=O)C(N=Nc3csc(C(N)=O)n3)C2SC1.[Li+] |
|  | Optimized (FFN) | CON=C(C(=O)NC1C(=O)N2C(C(=O)[O-])=C(CSc3nccs3)CSC12)C(C)O.[Na+] |
| Ceftazidime | Original | CC(C)(ON=C(C(=O)NC1C(=O)N2C(C(=O)[O-])=C(C[n+]3ccccc3)CSC12)c1csc(N)n1)C(=O)O |
|  | Optimized (GP) | CC(=O)OC(C(=O)O)C1C(=O)N2C(C(=O)O)=C(C[n+]3ccccc3)CSC12[CH2+][Cl+3] |
|  | Optimized (FFN) | Cn1cc(OC(=O)CF)c2c1C(O)C(NC(=O)CCl)=N2 |
| Minocycline Hydrochloride | Original | CN(C)c1ccc(O)c2c1CC1CC3C(N(C)C)C(O)=C(C(N)=O)C(=O)C3(O)C(O)=C1C2=O.Cl |
|  | Optimized (GP) | CN(C)Cc1cc(O)c2c(c1O)C(=O)C1=C(O)C(N(C)C)C3C(=O)C(O)(C(=O)C(O)=C(c4ccc(S)cc4)C3O)C(O)C1C2 |
|  | Optimized (FFN) | C#Cc1ccc2c(c1)-c1ccc(C)cc1C1=C(O)C(O)=C1C(C(C)C)N2CO.O |

**Table S6.** Properties of 8 sample compounds compared to corresponding seeds.

| Compound | Type | MW | Log P | HBD | HBA | TPSA | SAS |
| --- | --- | --- | --- | --- | --- | --- | --- |
| Cefazolin Sodium | Original | 476.501 | -4.96678 | 1 | 12 | 158.92 | 4.156638 |
|  | Optimized (GP) | 452.519 | -0.42678 | 1 | 10 | 141.35 | 4.078931 |
|  | Optimized (FFN) | 483.511 | -4.34378 | 1 | 11 | 146.03 | 4.048571 |
| Cefotaxime Sodium | Original | 477.456 | -4.9532 | 3 | 12 | 176.34 | 4.17153 |
|  | Optimized (GP) | 417.354 | -3.8195 | 2 | 11 | 167.44 | 4.720643 |
|  | Optimized (FFN) | 480.525 | -3.9737 | 2 | 10 | 144.25 | 4.677817 |
| Ceftazidime | Original | 546.587 | -1.2992 | 4 | 13 | 191.22 | 4.320142 |
|  | Optimized (GP) | 441.869 | -0.2201 | 2 | 9 | 125.09 | 4.961169 |
|  | Optimized (FFN) | 303.677 | 0.332 | 2 | 7 | 92.92 | 4.179883 |
| Minocycline Hydrochloride | Original | 493.944 | 0.0684 | 6 | 10 | 164.63 | 4.408552 |
|  | Optimized (GP) | 626.684 | 0.07492 | 7 | 12 | 199.3 | 2.572019 |
|  | Optimized (FFN) | 391.467 | 3.70782 | 5 | 5 | 95.43 | 3.758594 |

**Table S7.** SMILES of the corresponding molecule in Fig. S8.

| Seed | SMILES |
| --- | --- |
| Seed 1 | Cc1nnc(SCC2=C(C(=O)[O-])N3C(=O)C(NC(=O)Cn4cnnn4)C3SC2)s1.[Na+] |
|  | Cc1nnc(SCC2=C(C(=O)[O-])N3C(=O)C(NC(=O)CSc4nnn[nH]4)C3SC2)o1 |
|  | Cc1c[n+](CC2=C(C(=O)[O-])N3C(=O)C(NC(=O)CSc4nnns4)C3SC2)ccn1.O |
|  | Cc1nnc(SCC2=C(C(=O)[O-])N3C(=O)C(NC(=O)C(O)c4cscn4)C3SC2)n1CC(=O)[O-] |
|  | Cc1nnc(SCC2=C(C(=O)O)N3C(=O)C(NC(=O)Cc4cnc[n-]4)C3SC2)s1.[Na+] |
|  | Cc1nc(Cc2cccs2)c(CC(=O)O)c(=O)n1C1C(=O)N2C(C(=O)[O-])=C(CSc3nnnn3C)CSC12.[Na+] |
|  | Cc1nc2c(s1)c1c(c[n+]2[O-])nnn1CC1=C(C(=O)[O-])N2C(=O)C3NC(=O)C(OC(=O)C(=O)O)C3C2SC1 |
|  | Cn1cc[n+](CC2=C(C(=O)[O-])N3C(=O)C(NC(=O)C(OCn4cncn4)c4csc(Br)c4)C3SC2)n1 |
|  | Cc1nnc(SCC2=C(C(=O)[O-])N3C(=O)C(NC(=O)Cc4cnco4)C3SC2)o1 |
| Seed 2 | CON=C(C(=O)NC1C(=O)N2C(C(=O)[O-])=C(COC(C)=O)CSC12)c1csc(N)n1.[Na+] |
|  | CON=C(C(=O)NC1C(=O)N2C(C(=O)[O-])=C(COC(C)=O)CSC12)c1csc(C(C)=O)n1.[Cl] |
|  | CON=C(C(=O)[O-])C1=C(C(=O)OC(C)=O)N2C(=O)C(Nc3nccs3)C2SC1 |
|  | CON=C(C(=O)NC1C(=O)N2C(C(=O)[O-])=C(COC(C)=O)CSC12)c1csc([N+](=O)[O-])n1 |
|  | CON=C(C(=O)NC1C(=O)N2C(C(=O)OC(C)(C)C(O)C(=O)[O-])=CCSC12)c1csc(N)n1 |
|  | CON=C(C(=O)NC1C(=O)N2C(C(=O)[O-])=C(COC(C)=O)CSC12)c1csc(C)n1 |
|  | COC1(NC(C)=O)C(=O)N2C(C(=O)[O-])=C(CSc3nc[n+]([O-])cc3C=O)CSC(C)=C21 |
|  | CON=C(C(=O)NC1C(=O)N2C(C(=O)[O-])=C(COC(C)=O)CSC12)c1csc([O-])n1 |
|  | CON=C(C(=O)NC1C(=O)N2C(C(=O)[O-])=C(COC(C)=O)CSC12)c1cncs1.[Na+] |
| Seed 3 | CC(C)(ON=C(C(=O)NC1C(=O)N2C(C(=O)[O-])=C(C[n+]3ccccc3)CSC12)c1csc(N)n1)C(=O)O |
|  | CC(=O)OCC1=C(C(=O)O)N2C(=O)C(NC(=O)C(=NOC(Cn3cc[n+](C)c3)c3cccs3)c3csc(N)n3)C2SC1(C)C |
|  | CC(=O)OCC1=C(C(=O)O)N2C(=O)C(N=C(O)C(=O)c3csc(N)c3)C2SC1[AlH3]Cl |
|  | CC(=O)OCc1cccnc1NC1C(=O)N2C(C(=O)O)=C(CSc3cccnc3)CSC12 |
|  | CC(=O)OCc1cccnc1NC1C(=O)N2C(C(=O)O)=C(CSc3ncc(C)c[n+]3C)CSC12 |
|  | CC(=O)OCC1=C(C(=O)O)N2C(=O)C(NC(=O)C(=NOC(C)(C)c3csc(N)n3)c3ccccc3)C2SC1 |
|  | CC(=O)OC(C(=O)NC1C(=O)N2C(C(=O)[O-])=C(C[n+]3ccccc3)CSC12)c1csc(N)n1 |
|  | CC(=O)OC1C(=O)N2C(C(=O)O)=C(CNC(Cn3ccnc3)c3csc(=O)[nH]3)CSC12 |
| Seed 4 | CN(C)c1ccc(O)c2c1CC1CC3C(N(C)C)C(O)=C(C(N)=O)C(=O)C3(O)C(O)=C1C2=O.Cl |
|  | CC(C)=CCc1ccc(O)c2c1C(=O)C1=C(O)C3(O)C(=O)C(C(N)=O)=C(O)C(N(C)C)C3CC1C2O |
|  | CN(C)C1C(O)=C(C(N)=O)C(=O)C2(O)C(O)=C3C(=O)c4c(O)ccc(O)c4C(C)(C)C3CC12 |
|  | CN(C)C1C(=O)C(O)=C2C(=O)C3=C(O)c4c(Cl)ccc(C(N)=O)c4OC3C2C1(O)CCl |
|  | CN(C)C1C(O)=C(C(N)=O)C(=O)C2(O)C(O)=C3C(=O)c4c(ccc(Cl)c4O)C3CC12 |
|  | COc1ccc(C2CC3=C(C(=O)C(N(C)C)C(O)c4c(O)cc(C(N)=O)c(O)c43)C(O)C2)cc1O |
|  | CN(C)CC1C(O)=C(C(N)=O)C(=O)C2C(O)C3=C(O)c4c(ccc(C(C)(C)C)c4O)C(=O)C3C(O)C12 |
|  | CN(C)C1C(O)=C2C(=O)c3c(C4c5ccc(O)c(O)c5C(=O)C4N(C)C)ccnc3C2CC1O.Cl |

**Table S8.** SMILES of the corresponding molecule in Fig. S9.

| Seed | SMILES |
| --- | --- |
| Seed 1 | Cc1nnc(SCC2=C(C(=O)[O-])N3C(=O)C(NC(=O)Cn4cnnn4)C3SC2)s1.[Na+] |
|  | CC(=O)C1=C(C(=O)[O-])N2C(=O)C(NC(=O)C(CO)Sc3nncs3)C2SC1 |
|  | CON=C(C(=O)NC1C(=O)N2C(C(=O)[O-])=C(CSc3nnc(CC(=O)O)s3)CSC12)c1cn(C)cn1 |
|  | Cc1nnc(CSC2=C(C(=O)[O-])N3C(=O)C(NC(=O)C(O)c4cscn4)C3SC2)c(=O)[nH]1 |
|  | CC1=C(C(=O)[O-])N2C(=O)C(SCc3csc(Cn4cc(C(N)=O)nn4)n3)C2SC1 |
|  | Cc1cc2nc(C3=C(C(=O)[O-])N4C(=O)C(NC(=O)CSc5nnno5)C4SC3)csc-2n1.[Na+] |
|  | Cc1cc(=O)n2nc(C3SCCNC3C(=O)O)nc2s1 |
|  | Cc1cc(O)c2[nH]c(C(=O)C3CC(C(=O)O)NC3=O)nc2n1.[Na+] |
| Seed 2 | CON=C(C(=O)NC1C(=O)N2C(C(=O)[O-])=C(COC(C)=O)CSC12)c1csc(N)n1.[Na+] |
|  | CC(=O)O.CON=C(C(=O)NC1C(=O)N2C(C(=O)[O-])=C(COC(C)=O)CSC12)c1cn(C)cn1 |
|  | CON=C(C(=O)[O-])C1C(=O)N2C(C(=O)[O-])=C(COC(C)=O)CSC12 |
|  | CON=C(C(=O)NC1C(=O)N2C(C(=O)[O-])=C(CSCC(=O)O)CSC12)c1cncnc1N |
|  | CON=C(C(=O)[O-])c1csc(C2C=C(C(=O)[O-])C(C(N)=O)O2)c1O |
|  | CON=C(C(=O)[O-])C1C(=O)N2C(C(=O)[O-])=C(CSc3cncn3C)CSC12.[Cl] |
|  | CON1C=C(C(N)=O)C2=C(C(=O)O)C(O)C3=C(C(=O)CSC3)C21.[Na+] |
|  | C[Se]c1c(C#N)cn2c1C(=O)OCC2C(C)=O |
|  | C[Se]c1c(C(N)=O)c(C(C)=O)cn1C1COC(=O)C1[AlH3]O |
| Seed 3 | CC(C)(ON=C(C(=O)NC1C(=O)N2C(C(=O)[O-])=C(C[n+]3ccccc3)CSC12)c1csc(N)n1)C(=O)O |
|  | CC(=O)OCC1=C(C(=O)O)N2C(=O)C(NC(=O)C(O)c3csc(CSc4ccccc4)n3)C2SC1.[Na+] |
|  | CC(O)C1C(=O)N2C(C(=O)O)=C(COC(=O)C(C)(C)Sc3nc4cc(C(N)=O)c(N)cc4n3C)CSC12 |
|  | CC(=O)OCC1=CN2CCN(C)C2SC1.C[n+]1ccccc1.O=C([O-])C=C(O)C(=O)C(=O)NC(C(=O)O)c1csc(-c2ccccc2)n1.[Na+] |
|  | Cc1ccc[n+](CC(=O)NC2C(=O)N3C(C(=O)O)=C(COC(N)=O)CSC23)c1 |
|  | CC(=O)c1ccc[n+](CC(=O)NC2C(=O)N3C(C(=O)O)=C(CS)C23)c1 |
|  | Cc1ccc2c(C(=O)O)cn(CC(=O)NC3COC3)c2n1 |
|  | Cc1cc(CCO)nc(C(N)=O)c1 |
| Seed 4 | CN(C)c1ccc(O)c2c1CC1CC3C(N(C)C)C(O)=C(C(N)=O)C(=O)C3(O)C(O)=C1C2=O.Cl |
|  | CN(C)CC1c2c(O)ccc(O)c2C(=O)C2=C(O)C3(O)C(=O)C(O)=CC(N(C)C)C3CC21.Cl |
|  | CN(C)CC1c2occc2C(=O)C2=C(O)C3(O)C(=O)C(C(N)=O)=C(O)C(N(C)C)C3CC21.Cl |
|  | CN(C)c1ccc(Cl)c2c1CC1CC3C(N(C)C)C(O)=C(C(N)=O)C(=O)C3(O)C(O)C21 |
|  | CN(C)C1C(O)=C(C(N)=O)C(=O)C2(O)C(O)=C3C(=O)c4c(ccc(O)c4O)C(C)(C)C3CC12.Cl |
|  | CN(C)C1C(O)=C(C(N)=O)C(=O)C2(O)C(O)=C3C(=O)c4c(O)ccc(C(C)(C)C)c4C3CC12.Cl |
|  | CC(=O)c1ccc(N(C)C)c2c1C1C(=C(O)C(=O)C1O)C2=O.Cl |
|  | C#Cc1ccc2c(c1)C(O)n1c[n+](C)cc1C2=O |
